# Metabolite toxicity determines the pace of molecular evolution within microbial populations

**DOI:** 10.1186/s12862-017-0906-2

**Published:** 2017-02-14

**Authors:** Elin E. Lilja, David R. Johnson

**Affiliations:** 1 Department of Environmental Microbiology, Eawag, Überlandstrasse 133, 8600 Dübendorf, Switzerland; 2 Department of Environmental Systems Science, ETH Zürich, Zürich, Switzerland

**Keywords:** Experimental evolution, Denitrification, Nitrite toxicity, Microbial populations, Molecular evolution

## Abstract

**Background:**

The production of toxic metabolites has shaped the spatial and temporal arrangement of metabolic processes within microbial cells. While diverse solutions to mitigate metabolite toxicity have evolved, less is known about how evolution itself is affected by metabolite toxicity. We hypothesized that the pace of molecular evolution should increase as metabolite toxicity increases. At least two mechanisms could cause this. First, metabolite toxicity could increase the mutation rate. Second, metabolite toxicity could increase the number of available mutations with large beneficial effects that selection could act upon (*e.g*., mutations that provide tolerance to toxicity), which consequently would increase the rate at which those mutations increase in frequency.

**Results:**

We tested this hypothesis by experimentally evolving the bacterium *Pseudomonas stutzeri* under denitrifying conditions. The metabolite nitrite accumulates during denitrification and has pH-dependent toxic effects, which allowed us to evolve *P. stutzeri* at different magnitudes of nitrite toxicity. We demonstrate that increased nitrite toxicity results in an increased pace of molecular evolution. We further demonstrate that this increase is generally due to an increased number of available mutations with large beneficial effects and not to an increased mutation rate.

**Conclusions:**

Our results demonstrate that the production of toxic metabolites can have important impacts on the evolutionary processes of microbial cells. Given the ubiquity of toxic metabolites, they could also have implications for understanding the evolutionary histories of biological organisms.

**Electronic supplementary material:**

The online version of this article (doi:10.1186/s12862-017-0906-2) contains supplementary material, which is available to authorized users.

## Background

**Fig. 1:**
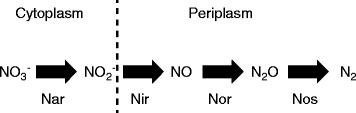
The denitrification pathway of *P. stutzeri*. Nitrate is first reduced to nitrite in the cytoplasm. Nitrite is then actively transported to the periplasmic space where it is further reduced through several intermediates to dinitrogen gas. The consequence is that nitrite accumulates in the periplasm. Enzymes: Nar, nitrate reductase; Nir, nitrite reductase; Nor, nitric oxide reductase; Nos, nitrous oxide reductase

The production of toxic metabolites is an important factor that shapes the spatial and temporal arrangement of metabolic processes within microbial cells [1]. Toxic metabolites may form as intermediates, side products, or end products of metabolism, and there are numerous examples of how microbial cells arrange metabolic processes in space and time to prevent the accumulation of these metabolites or to divert them away from sensitive biological processes [1]. Consider the denitrification process whereby some types of microbial cells sequentially reduce nitrate (NO_3_^−^) to nitrite (NO_2_^−^), nitric oxide (NO), nitrous oxide (N_2_O) and finally to dinitrogen gas (N_2_) to yield energy (Fig. 1) [2]. The metabolic intermediate nitric oxide is a free radical that has cytotoxic effects on cell division and forms metal-nitrosyl complexes with enzymes [3, 4]. Indeed, nitric oxide performs many of the antimicrobial functions of macrophages [5]. Thus, denitrifying microorganisms must maintain nitric oxide at low concentrations during denitrification. To achieve this, nitric oxide regulates the transcription of denitrification genes [4], suggesting regulatory solutions to control the production and consumption rates of nitric oxide and prevent its accumulation. In addition, denitrifying microorganisms typically produce and consume nitric oxide in the periplasmic space of the cell, thus preventing nitric oxide from interacting with sensitive biological processes that occur within the cytoplasm [6].

While it is clear that the production of toxic metabolites have shaped the spatial and temporal arrangement of metabolic processes within microbial cells, it is less clear how metabolite toxicity affects the pace of molecular evolution itself. In this context, we use the term “pace of molecular evolution” to refer to the number of mutations that accumulate per generation and not to the number of mutations that accumulate per unit time. Metabolite toxicity could affect the pace of molecular evolution by at least two distinct mechanisms. First, metabolite toxicity might directly (*e.g*., acting on DNA) or indirectly (*e.g*., via general stress response) increase the mutation rate, thus providing a larger set of mutations for selection or drift to act upon [7, 8]. Second, metabolite toxicity might decrease the absolute fitness of the cell; that is, metabolite toxicity might cause slower growth rates or lower yields due to their potentially deleterious effects on biological processes. This could result in increased selection pressure and increased numbers of available mutations with large beneficial effects for selection to act upon (*e.g*., mutations that provide tolerance to or eliminate metabolite toxicity), thus increasing the rate at which those mutations increase in frequency [9, 10].

Our main objectives were to experimentally test whether the toxicity of a single metabolite does indeed increase the pace of molecular evolution and to determine by which mechanisms such an effect might emerge (*i.e*., by increasing the mutation rate or by increasing the number of available mutations with large beneficial effects for selection to act upon). To address these objectives, we developed an experimental system where we could manipulate the toxicity of a single metabolite. We then experimentally evolved replicated populations of bacteria at two distinct levels of metabolite toxicity, quantified the types and numbers of mutations that accumulated during experimental evolution, and tested whether metabolite toxicity affects the pace of molecular evolution.

Our experimental system is based on the denitrifying bacterium *Pseudomonas stutzeri* A1501 (hereafter referred to as *P. stutzeri*), which is a facultative anaerobe with a fully sequenced genome [11, 12]. In the absence of oxygen, *P. stutzeri* can use nitrogen oxides as terminal electron acceptors to support its growth [13]. *P. stutzeri* sequentially reduces nitrate (NO_3_^−^) to nitrite (NO_2_^−^), nitric oxide (NO), nitrous oxide (N_2_O), and finally to dinitrogen gas (N_2_) using different enzyme complexes for each reduction step (Fig. 1) [6]. An important feature of this experimental system is that the metabolite nitrite accumulates in batch culture and has pH-dependent toxic effects [14, 15]. As the pH decreases, nitrite increasingly generates nitrous acid (HNO_2_), which uncouples proton translocation [16, 17]. In addition, nitrite increasingly and spontaneously generates nitric oxide radicals that impose cytotoxic effects on cell division and form metal-nitrosyl complexes with enzymes [3]. The consequence is that, as the pH decreases, the increased toxicity of nitrite has negative effects on growth and metabolic activity [15, 18]. In general, nitrite toxicity has negligible effects at pH 7.5 and severe effects at pH 6.5, while pH itself has no measureable effects under the same pH range [15]. The pH of the culture medium can therefore be used to manipulate nitrite toxicity without creating substantial confounding factors [15], thus allowing us to test the hypothesis that increased nitrite toxicity increases the pace of molecular evolution. Indeed, we previously demonstrated that pH itself has no statistically significant effects on the growth of *P. stutzeri* under the experimental conditions used in this study while nitrite is non-toxic at pH 7.5 but severely toxic at pH 6.5 [15].

## Results

### Increased nitrite toxicity accelerates molecular evolution

**Fig. 2:**
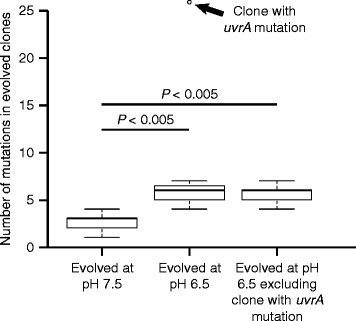
The number of mutations that accumulated in clones after evolution at pH 6.5 (strong nitrite toxicity) or pH 7.5 (weak nitrite toxicity). Horizontal bars and *P*-values indicate the outcomes of two-sample Wilcoxon rank-sum tests. The arrow indicates the clone with a mutation in *uvrA*. Data are presented as Tukey box-plots

Our first objective was to experimentally test whether increased toxicity of a single metabolite, nitrite (NO_2_^−^), increases the pace of molecular evolution. To achieve this objective, we experimentally evolved eight populations of *P. stutzeri* at pH 6.5 (strong nitrite toxicity) and eight populations at pH 7.5 (weak nitrite toxicity) for approximately 700 generations. We then randomly selected a single clone from each evolved population, sequenced its genome, and quantified the number of mutations within that clone when compared to the ancestral clone (Additional file 1: Table S1-2). We found that more mutations accumulated in clones evolved at pH 6.5 than at pH 7.5, thus supporting our main hypothesis (Wilcoxon rank-sum test; Bonferroni-corrected *P* < 0.01, n_1_ = n_2_ = 8) (Fig. 2). We further found that one clone evolved at pH 6.5 accumulated five times more mutations than the other clones evolved at pH 6.5. This clone contains a loss of function mutation in the *uvrA* gene, which encodes for subunit A of excinuclease, a protein involved in nucleotide excision-based DNA repair [19, 20]. Thus, this mutation likely caused an increased mutation rate in this clone. We therefore further tested whether more mutations accumulated in clones evolved at pH 6.5 than at pH 7.5 when this clone was removed from the statistical analysis, and this was indeed the case (Wilcoxon rank-sum test; Bonferroni-corrected *P* < 0.01, n_1_ = 8, n_2_ = 7) (Fig. 2). This was also true when we only took into account non-synonymous mutations within protein coding regions (Additional file 1: Figure S1). Thus, our data supports the hypothesis that the increased reactivity of a single metabolite can increase the pace of molecular evolution.

**Fig. 3:**
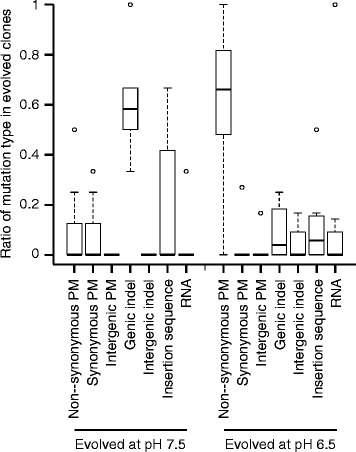
The types of mutations that accumulated in clones after evolution at pH 6.5 (strong nitrite toxicity) or pH 7.5 (weak nitrite toxicity). Each mutation was categorized by type and frequency among all mutations. Data are presented as Tukey box-plots

Increased mutation rates are unlikely (but not unequivocally excluded) to account for the increased pace of molecular evolution. The increased numbers of mutations in clones evolved at pH 6.5 (strong nitrite toxicity) could be due to either increased mutation rates or increased numbers of available mutations with large beneficial effects that selection could act upon. They could also be caused by epistatic effects among mutations or by demographic differences between treatments. We investigated whether increased mutation rates were important by categorizing all mutations by mutation type: point mutations (non-synonymous, synonymous or intergenic), indels (genic and intergenic), large deletions (>250 bp), mutations conferred by insertion sequences, and mutations in ribosomal genes (Fig. 3). We reasoned that, if the accelerated pace of molecular evolution was predominantly caused by increased mutation rates, than we would expect increased numbers of synonymous substitutions to accumulate within those clones. This was not the case. There was no significant difference in the frequency of synonymous substitutions (Wilcoxon rank-sum test, *P* > 0.5, n_1_ = n_2_ = 8) or the absolute abundance of synonymous substitutions (Wilcoxon rank-sum test, *P* > 0.6, n_1_ = n_2_ = 8) for clones evolved at pH 6.5 compared to clones evolved at pH 7.5 (Fig. 3). This was true regardless of whether we included or removed the clone with the mutation in the *uvrA* gene from the analysis. Indeed, we did not detect any synonymous mutations at all in the clones evolved at pH 6.5, except within the clone that has a mutation within the *uvrA* gene. Taken together, with the exception of the single clone with a mutation in the *uvrA* gene, we have no evidence that differences in mutation rates generally explain the increased numbers of mutations that accumulated within clones evolved at pH 6.5 (strong nitrite toxicity). However, we acknowledge that we do not have direct measures of mutation rates, and we instead rely on proxy measures such as comparing rates of synonymous substitutions. It is possible that the absence of different numbers of synonymous substitutions may be due to the low numbers of total mutations that were observed in this study, which could be addressed in the future via sequencing additional clones or investigating longer evolutionary time-scales.

Interestingly, we did find that clones evolved at pH 6.5 (strong nitrite toxicity) accumulated different types of mutations than those evolved at pH 7.5 (weak nitrite toxicity). Clones evolved at pH 6.5, which exhibited the increased pace of molecular evolution, accumulated significantly more non-synonymous substitutions than other types of mutations (ANOVA followed by Tukey’s HSD post hoc analysis; *P* < 0.01) (Fig. 3). In contrast, clones evolved at pH 7.5 accumulated significantly more short indels in coding regions than other types of mutations (ANOVA followed by Tukey’s HSD post hoc analysis; *P* < 0.01) (Fig. 3). The different types of mutations did not always occur in different target genes. For example, mutations occurred in the *oprQ* gene at both pH conditions, but the mutations are short deletions at pH 7.5 and primarily single-base substitutions at pH 6.5.

### Increased nitrite toxicity leads to larger increases in absolute fitness

**Fig. 4:**
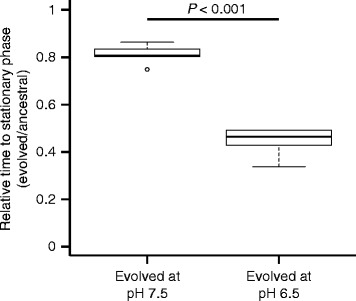
Relative time to stationary phase for each experimental evolution condition. Data are the time to reach stationary phase for the evolved clones divided by the time to stationary phase for the ancestral clones. The horizontal bar and *P*-value indicates the outcome of a two-sample Wilcoxon rank-sum test. The asterisk indicates *P* < 0.05. The data are presented as Tukey box-plots

If the increased numbers of mutations in clones evolved at pH 6.5 (strong nitrite toxicity) were generally due to increased numbers of available mutations with large beneficial effects that selection could act upon, then we would expect the populations evolved at pH 6.5 to have larger increases in fitness than the populations evolved at pH 7.5 relative to the ancestral clones. We tested this expectation using growth assays and measuring the time for populations to reach stationary phase, which is equivalent to the time required for complete substrate consumption under our experimental conditions [15]. We calculated the relative time for complete substrate consumption as the time for the evolved populations to enter stationary phase (t_evol_) divided by the time for the ancestral populations to enter stationary phase (t_anc_). We report the ratios of t_evol_ to t_anc_ in Fig. 4. We found that the populations evolved at pH 6.5 had significantly smaller ratios of t_evol_ to t_anc_ than those evolved at pH 7.5 (Wilcoxon rank-sum test; *P* < 0.001, n_1_ = n_2_ = 8) (Fig. 4). Overall, the populations evolved at pH 6.5 required approximately 50% of the ancestral time to reach stationary phase while the populations evolved at pH 7.5 required about 80% of the ancestral time (Fig. 4). We note that the clone with the mutation in the *uvrC* gene is not an outlier for these analyses, suggesting that the increased number of mutations resulting from a potentially increased mutation rate does not cause a measurable change in the fitness of this clone. Together, our results are consistent with the increased numbers of mutations that accumulated in populations evolved at pH 6.5 (strong nitrite toxicity) being caused by increased numbers of available mutations with large beneficial effects.

### Increased nitrite toxicity leads to larger increases in competitive fitness

Because evolution at pH 6.5 (high nitrite toxicity) led to both increased numbers of mutations and larger increases in fitness, we further investigated whether evolution at pH 6.5 also results in increased competitive fitness. We define competitive fitness as the relative fitness of an evolved clone when competed directly against its ancestor in co-culture. To address this, we performed four different competition assays where we competed evolved clones against ancestral clones. We initiated the competition assays with 5% of evolved cells and 95% of ancestral cells, as our previous competition assays using an initial ratio of 50% of evolved cells and 50% of ancestral cells did not yield any significant differences (Additional file 1: Figure S2). This was likely because the evolved cells accumulated less nitrite. Thus, when they are present at high frequencies, they reduce nitrite accumulation and “rescue” the ancestral cells.

**Fig. 5:**
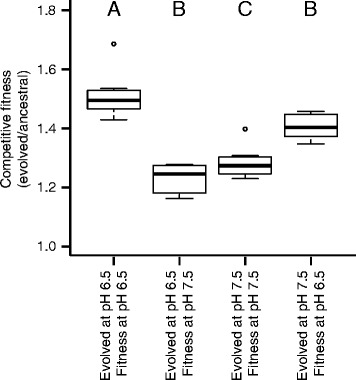
Competitive fitness of evolved clones relative to the ancestor. Evolved cells were initially present in the culture at a frequency of 5%. Differences were compared using an ANOVA test followed by a post hoc Tukey’s HSD test. Significant differences correspond to *P* < 0.01. Alphabetic assignments indicate groups that are statistically different from each other

We first tested whether the clones evolved at pH 6.5 (strong nitrite toxicity) improved in competitive fitness more than the clones evolved at pH 7.5 (weak nitrite toxicity), which would be expected given that the clones evolved at pH 6.5 accumulated more mutations and consumed substrates more rapidly as compared to the ancestor (i.e., they required less time to reach stationary phase). To accomplish this, we compared the competitive fitness of the evolved clones when they were competed against the ancestor at the same conditions as they were evolved. We found that the clones evolved at pH 6.5 increased in competitive fitness significantly more than the clones evolved at pH 7.5 (ANOVA with a Tukey’s HSD post hoc analysis, *P* < 0.01) (Fig. 5; first boxplot compared to the third boxplot), thus supporting our expectation.

We next assessed competitive fitness in the non-selected environments. First, we compared the competitive fitness of clones evolved at pH 7.5 (weak nitrite toxicity) when competed against the ancestor at pH 6.5 (strong nitrite toxicity) or pH 7.5. The objective was to investigate if the fitness benefits acquired during evolution at pH 7.5 become more pronounced at pH 6.5. We found that the clones evolved at pH 7.5 increased in competitive fitness significantly more when competed at pH 6.5 than at pH 7.5 (ANOVA with a Tukey’s HSD post hoc analysis, *P* < 0.01) (Fig. 5; last boxplot compared to the third boxplot). This indicates that the mutations that accumulated in clones evolved at pH 7.5 have larger benefits at pH 6.5 than at pH 7.5. This could occur, for example, if mutations emerged that reduce the intracellular accumulation of nitrite. While potentially beneficial regardless of the pH, the beneficial effects would be greater at pH 6.5 when nitrite is toxic. Further experiments would be needed to test this possibility.

Next, we compared the competitive fitness of clones evolved at pH 6.5 (strong nitrite toxicity) or pH 7.5 (weak nitrite toxicity) against the ancestor at pH 6.5. The objective was to investigate if evolution at pH 6.5 leads to larger fitness benefits at pH 6.5 than evolution at pH 7.5. Indeed, we found that the clones evolved at pH 6.5 increased in competitive fitness significantly more than the clones evolved at pH 7.5 (ANOVA with a Tukey’s HSD post hoc analysis, *P* < 0.01) (Fig. 5; first boxplot compared to the last boxplot). This indicates that, even though the mutations accumulated in clones evolved at pH 7.5 had larger benefits at pH 6.5 than at pH 7.5, this is not sufficient to account for all of the increased fitness of clones at pH 6.5.

Finally, we compared the competitive fitness of the clones evolved at pH 6.5 (strong nitrite toxicity) or pH 7.5 (weak nitrite toxicity) when competed against the ancestor at pH 7.5. The objective was to investigate if the increased fitness benefits in clones evolved at pH 6.5 have any significant effect at pH 7.5. We found that there is no significant difference in the competitive fitness between the two evolutionary conditions when assayed at pH 7.5 (ANOVA with a Tukey’s HSD post hoc analysis, *P* > 0.01) (Fig. 5; second boxplot compared to the third boxplot). These results indicate that there are indeed mutations that have a beneficial effect on fitness at pH 6.5 that have no effect at pH 7.5. Taken together with the outcomes from the other competition assays, our data supports the idea that the larger increase in competitive fitness found after evolution at pH 6.5 is both due to an increased benefit of the same type of mutations that are found at pH 7.5 as well as additional types of mutations that have a benefit only at pH 6.5. We acknowledge, however, that interactions between mutations may also be critical in addition to individual mutation effects.

### Increased nitrite toxicity selects for mutations in genes with a broader variety of functional annotations

**Fig. 6:**
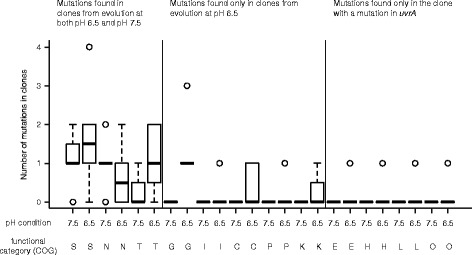
Functional categorization of genes that contain mutations in evolved clones for each experimental evolution condition. Each mutation was categorized by type. Data are presented as Tukey box-plots. Functional categories (COG): S, function unknown; N, cell motility; T, signal transduction; G, carbohydrate transport and metabolism; I, lipid transport and metabolism; C, energy production and conversion; P, inorganic ion transport and metabolism; K, transcription; E, amino acid transport and metabolism; H, coenzyme transport and metabolism; L, replication, recombination and repair; O, posttranslational modification, protein turnover, and chaperones

We next tested whether evolution at pH 6.5 (strong nitrite toxicity) not only promotes more mutations to accumulate, but also affects the functional composition of those mutations. To address this, we functionally categorized each gene with a mutation according to existing annotations [11, 21]. In this analysis, we only included non-synonymous substitutions in coding regions or point mutations in intergenic regions that are likely to be involved in transcription (Fig. 6). Clones evolved at pH 7.5 only have mutations in three functional categories: cell motility, signal transduction and unknown functions. Mutations in these types of functions are also commonly found in clones evolved at pH 6.5. The numbers of mutations in the clones do not differ between the two conditions (strong or weak nitrite toxicity) for these functional categories. Clones evolved at pH 6.5 had mutations in all of the same categories discussed above, but also have mutations in additional categories: carbohydrate transport and metabolism, lipid transport and metabolism, energy production and conversion, inorganic ion transport and metabolism and transcription. However, the only functional category where this is statistically significant from the clones evolved at pH 7.5 is carbohydrate transport and metabolism (Wilcoxon rank sum test, n_1_ = n_2_ = 8, *P* < 0.001). Indeed, each of the clones evolved at pH 6.5 have a mutation in at least one gene encoding for an enzyme involved in carbohydrate metabolism, while none of the clones evolved at pH 7.5 have a mutation in a gene involved in carbohydrate metabolism. The other functional categories are those where mutations were only found in the clone with a mutation in *uvrA* (Fig. 6). A few clones evolved at pH 6.5 also have mutations in genes involved in denitrification that were not observed in clones evolved at pH 7.5 (denitrification-related genes are distributed among a variety of functional categories). In conclusion, evolution at pH 6.5 (strong nitrite toxicity) led not only to the accumulation of more mutations but also to the accumulation of mutations with increased diversity of functional annotations.

### Populations evolved at increased nitrite reactivity have decreased efficiency in the use of alternative carbon substrates

We finally tested whether the increased numbers of mutations in populations evolved at pH 6.5 (strong nitrite toxicity) correlates with increased pleiotropic effects under non-selected environments. To accomplish this, we measured the performance of the ancestral and all of the evolved populations when growing on a variety of alternative carbon sources using Biolog PM1 plates. These plates contain 95 individual carbon sources and can be used to measure growth over time. We measured performance as the area under the growth curve (OD_600_ vs time) for each carbon substrate as described elsewhere [22]. We only took into account those carbon sources for which there was visible growth.

**Fig. 7:**
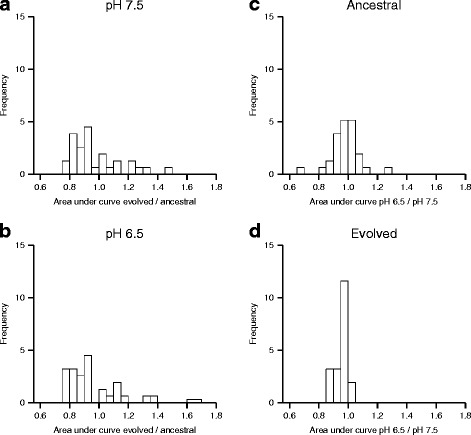
Performance of evolved and ancestral clones to utilize alternative carbon substrates. Carbon utilization was measured for 95 different carbon substrates. Performance was quantified as the area under the growth curve for each individual carbon substrate (OD_600_ *vs.* time), which takes into account both growth rate and yield. Data are presented as histograms. (**a**) Comparison of clones evolved at pH 7.5 with ancestral populations at pH 7.5; (**b**) comparison of clones evolved at pH 6.5 with ancestral clones at pH 6.5; (**c**) comparison of ancestral clones at pH 6.5 with ancestral clones at pH 7.5; (**d**) comparison of clones evolved at pH 6.5 with clones evolved at pH 7.5

First, we compared the populations evolved at pH 6.5 (strong nitrite toxicity) with the ancestor grown at pH 6.5 and the populations evolved at pH 7.5 (weak nitrite toxicity) with the ancestor grown at pH 7.5 (Fig. 7a-b). We found that populations evolved at pH 7.5 had no significant overall change in their ability to use alternative carbon substrates when compared to the ancestor; the average of evolved_area_/ancestral_area_ (area = area under curve) does not deviate from one (Fig. 7a; one sample Wilcoxon rank-sum test; *P* > 0.2). In contrast, populations evolved at pH 6.5 had an overall decrease in carbon source utilization; the average of evolved_area_/ancestral_area_ is significantly lower than one (Fig. 7b; one-sample Wilcoxon rank -sum test; *P* < 0.05). We also calculated the differences between the two evolution conditions for both the ancestors and the evolved populations; that is, we calculated the average at pH 6.5 and divided it by the average at pH 7.5 for each carbon source for the ancestors and for the evolved populations (Fig. 7c-d). The average of pH6.5_area_/pH7.5_area_ does not deviate from one for the ancestors, indicating that pH itself has no effect (Fig. 7c; one-sample Wilcoxon signed-rank test, *P* > 0.3). In contrast, the average of pH6.5_area_/pH7.5_area_ is significantly lower than one for the evolved populations, indicating that evolution at pH 6.5 does indeed have negative consequences on the utilization of non-selected substrates (Fig. 7d; one-sample Wilcoxon signed-rank test, *P* < 0.00001). Taken together, our data support the conclusion that the increased accumulation of mutations at pH 6.5 led to increased antagonistic pleiotropic effects in non-selected environments. This is likely due to the increased accumulation of mutations in carbohydrate utilization and transport genes at pH 6.5, which may have negative effects on the ability of those evolved populations to utilize alternative carbon substrates.

## Discussion

Our results are consistent with the hypothesis that increased toxicity of a single metabolic intermediate can accelerate the pace of molecular evolution, and this leads to larger increases in both absolute and relative fitness. We conclude that the increased rate of evolution is most likely due to the increased availability of mutations with large beneficial effects that selection can act upon rather than an increase in the mutation rate in response to stressful conditions, the latter of which has been repeatedly observed [7, 8, 23, 24]. This is based on evidence that there is no increase in synonymous mutations at strong nitrite toxicity (Fig. 3) and further supported by the fact that the additional mutations that accumulated during evolution at strong nitrite toxicity do not confer a significant additional increase in fitness at weak nitrite reactivity (Fig. 5). Furthermore, while we identified a single clone with five-fold more mutations than the other clones (Fig. 2), this clone did not show improved fitness when compared to the other clones. We note here that differences in pH may have also contributed towards the different outcomes, but we believe this is unlikely given that differences in pH over the experimentally evolved conditions have no observable effect on the growth of *P. stutzeri* [15].

The larger increases in fitness observed after evolution at strong nitrite toxicity were due to two mechanisms. The first mechanism is that the fitness benefits of mutations in genes encoding for certain functions had larger beneficial effects as nitrite toxicity increased. We base this conclusion on the fact that clones evolved at weak nitrite toxicity share mutations in genes with similar functions with clones evolved at strong nitrite toxicity, including mutations in genes involved in cell motility (*e.g.* *flgD*, *fleQ*), signal transduction (*phoP*), and in genes encoding for membrane proteins (*oprE3*, PST_2380 [a porin]) (Additional file 1: Table S1-2). This suggests that there are adaptations common to both conditions. Yet, the clones evolved at weak nitrite toxicity have larger increases in competitive fitness when grown at high nitrite toxicity than at low nitrite toxicity (Fig. 5), suggesting that the adaptations common to both conditions have larger benefits at stronger nitrite toxicity. This idea that the benefits of mutations can be larger (and thus requiring fewer generations to accumulate within the population) in a less fit background is consistent with several previous studies. For example, a recent study with yeast demonstrated that beneficial mutations have larger benefits in more stressful backgrounds, where the initial fitness was determined by the genotype rather the environment [25]. As another example, mutations conferring antibiotic resistance have larger benefits at higher antibiotic concentrations [26]. Thus, in the case of nitrite toxicity, mutations in certain functions have beneficial effects at both weak and strong nitrite toxicity, but those benefits increase as toxicity increases.

The second mechanism is that mutations emerged that are only beneficial at strong nitrite toxicity. That is, populations evolved at strong nitrite toxicity have larger increases in competitive fitness than populations evolved at weak nitrite toxicity when competed at strong nitrite toxicity (Fig. 5). Additionally, populations evolved at strong nitrite toxicity have no significant difference in the increase of competitive fitness compared to populations evolved at weak nitrite toxicity when competed at weak nitrite toxicity (Fig. 5). This idea is further supported by the fact that mutations in genes encoding for proteins involved in carbon metabolism (*PykA*, *Fbp*, *Gap-2*) and in denitrification were only identified in clones evolved at strong nitrite toxicity (Additional file 1: Table S1-2). These results indicate that mutations in functions such as carbon metabolism and denitrification only have beneficial effects at strong nitrite toxicity.

Our data additionally demonstrates that increased nitrite toxicity not only accelerates molecular evolution, but also reduces the niche breadth of the cells. We found that the increase in the number of mutations during evolution at strong nitrite toxicity led to increased antagonistic pleiotropic effects in non-selected environments, as demonstrated by the decrease in utilization efficiency of alternative carbon sources (Fig. 7). While statistically significant, we caution that these results may not be biologically significant, as the effects are typically small for many of the alternative carbon sources. However, the fact that all lineages evolved at strong nitrite toxicity have mutations in important enzymes involved in glycolysis and gluconeogenesis supports the idea that carbon utilization would be affected in these lineages. This suggests that evolution at increased nitrite toxicity leads to increased metabolic specialization and decreased niche breadth. This decrease in niche breadth may not be unexpected, as there is some evidence supporting the idea that adaptation to more stressful environments leads to increased trade-offs in non-selected environments [27]. Given the large numbers of substrates and intermediates that are present in the natural environment, metabolite toxicity may therefore be an important factor that limits niche breadth and, in turn, promotes biodiversity and the coexistence of different metabolically specialized genotypes. It could therefore provide a partial explanation for the extraordinary levels of microbial biodiversity present in many environments.

## Conclusions

Our results demonstrate that toxic metabolites can have important impacts on the evolutionary processes of microbial cells. Given the ubiquity of toxic metabolites, they have implications for understanding the evolutionary histories of biological organisms. Finally, because an increased pace of evolution correlates with reduced niche breadth, the production of toxic metabolites may help to explain the enormous amount of microbial diversity in the natural environment.

## Methods

### Strains and genetic manipulations

We obtained the wild-type bacterium *P. stutzeri* A1501 from the Biological Resource Center of Institut Pasteur (Paris, France) and used this strain to construct all of the *P. stutzeri* mutant strains described in this study (Supplementary Table S3). We introduced DNA fragments containing the isopropyl-β-D-thiogalactopyranosid (IPTG)-inducible P_*lac*_ promoter located immediately upstream of the *egfp* or *echerry* gene [28] into *P. stutzeri* A1601. These genes encode green or red fluorescent proteins, respectively. We introduced the DNA fragments using derivatives of the mini-Tn7T-LAC-Gm transposon and the pUC18T conditionally replicative delivery plasmid (Additional file 1: Table S3) as described elsewhere [29, 30]. Briefly, we constructed the derivative transposons by first purifying the pUC18T-mini-Tn7T-LAC-Gm plasmid from an overnight culture of *Escherichia coli* DH5α/λpir (Additional file 1: Table S3) [31]. We next PCR amplified the *egfp* or *echerry* gene using GoTaq DNA polymerase (Promega, Madison, WI, USA) and the oligonucleotide primers listed in Supplementary Table S4. These primers contain the *Bam*HI and *Kpn*I restriction sites that we used to clone the PCR products into the pUC18T-mini-Tn7T-LAC-Gm plasmid. We then digested the pUC18T-mini-Tn7T-LAC-Gm plasmid and the PCR products with *Bam*HI and *Kpn*I (Thermo Fisher Scientific, Waltham, MA, USA) and ligated the PCR products into the pUC18T-mini-Tn7T-LAC-Gm plasmid. We designated the assembled derivative plasmids as pUC18T-mini-Tn7T-LAC-Gm-*egfp* and pUC18T-mini-Tn7T-LAC-Gm-*echerry* respectively (Additional file 1: Table S3). We replicated the assembled derivative plasmids in *E. coli* DH5α/λpir (Additional file 1: Table S3) [31].

We used conjugative four-parental mating to deliver the assembled pUC18T-mini-Tn7T-LAC-Gm derivative plasmids along with the helper pUX-BF13 plasmid that expresses the transposase gene into *P. stutzeri* A1601 (Additional file 1: Table S1) as described elsewhere [30]. We selected *P. stutzeri* exconjugants by plating on 3-(*N*-morpholino)propanesulfonic acid (MOPS) agar plates containing 0.2% of sodium citrate and 10 μg ml^−1^ of gentamycin [30]. We verified plasmid segregation by testing for ampicillin sensitivity on lysogeny broth (LB) agar plates containing 100 μg ml^−1^ of ampicillin. We did not perform FRT excision of the gentamycin resistance marker [30] and all of the *P. stutzeri* mutant strains therefore retained gentamycin resistance (Additional file 1: Table S3). This was intentional to prevent contamination during the evolution experiment.

### Culture conditions

We cultured all *P. stutzeri* strains under aerobic conditions in a defined asparagine-citrate synthetic medium (ACS medium) [32] with 10 μg ml^−1^ of gentamicin. We cultured all *P. stutzeri* strains under anaerobic conditions in dinitrogen gas (N_2_)-sparged ACS medium amended with 10 mM of sodium nitrate (NaNO_3_) and 10 μg ml^−1^ of gentamycin. We reported a complete description of the methods to prepare and inoculate dinitrogen gas-sparged ACS medium elsewhere [15]. We incubated all *P. stutzeri* cultures at 30 °C with shaking at 220 rpm. The maximum observed nitrite concentration under these conditions was approximately 7 mM and the pH did not substantially change over the course of batch growth [15]."

### Evolution experiment

We experimentally evolved four populations that carry the *egfp* gene and four populations that carry the *echerry* gene for each pH condition (pH 6.5 or 7.5) for a total of 16 populations. This design controlled for any differences in the metabolic costs of carrying the *egfp* or *echerry* gene. Furthermore, we did not add IPTG to the medium during experimental evolution to avoid the metabolic cost of expressing the fluorescent proteins, and to therefore minimize the probability of selecting for loss-of-function mutations in the *egfp* or *echerry* gene over the course of the evolution experiment. We used the *egfp* and *echerry*-expressing traits to distinguish different strains during competition assays after experimental evolution (see below) and to periodically assess for cross-contamination between populations. For the latter test, we plated a small aliquot of each population onto an individual LB agar plate containing 0.1 mM of IPTG. We never observed cross-contamination during the experiment (*i.e*., we never observed populations that contained both *egfp* and *echerry*-expressing clones).

For the evolution experiment, we serially transferred batch cultures containing dinitrogen gas (N_2_)-sparged ACS medium amended with 10 mM of sodium nitrate (NaNO_3_) as the growth-limiting substrate. We transferred each population after entering stationary phase at a dilution of 1:200 (vol:vol) (although see the exceptions below) into fresh dinitrogen gas (N_2_)-sparged ACS medium for a total of 700 generations. We transferred the populations evolved at pH 7.5 (weak nitrite toxicity) every day, with some exceptions during the beginning of the evolution experiment when growth was slow or highly variable (*i.e*., some cultures grew slower or had long lag periods before growth was observed). We transferred the populations evolved at pH 6.5 (strong nitrite toxicity) every fourth day during the beginning of the evolution experiment and every second day at the end of the evolution experiment. In general, we performed each transfer at a 1:200 dilution (vol:vol), with some exceptions at the beginning of the evolutionary experiments when growth was slow or highly variable. At these times, we performed each transfer at a 1:50 or a 1:100 dilution (vol:vol) and we took these exceptions into account when estimating the total number of generations. We estimated the number of generations based on the dilution, where 7.64 generations could occur from a 1:200 (vol:vol) dilution (*i.e*., 2^7.64^ = 200). This estimation is appropriate if the cultures are allowed to completely consume all of the provided nitrogen oxides prior to transfer, which was the case for our experiment. We monitored optical density at 600 nm (OD_600_) to verify that the cultures had entered stationary phase, and thus consumed all the nitrogen oxides, prior to transfer.

### Genome sequencing

We streaked each evolved and ancestral population onto LB agar plates containing 10 μg ml^−1^ of gentamicin and 0.1 mM of IPTG and picked a single colony from each population. We grew the single clones in LB overnight and extracted genomic DNA with the Wizard Genomic DNA purification kit (Promega, Madison, WI). We sequenced genomic DNA using an Illumina HiSeq 2000 sequencer (Illumina, San Diego, CA) with 100 cycles of paired-end sequencing. We performed primary data analysis, de-multiplexing, and quality control analyses of the sequencing data using FastQC (Illumina, San Diego, CA). We performed additional quality filtering of the raw reads, removed duplicate reads, and trimmed ambiguous base pairs using PRINSEQ-lite v0.20.4 [33]. Finally, we identified differences between the evolved genomes and the ancestral genome using Breseq v.0.24rc5 as described elsewhere [34]. These genetic differences included synonymous and non-synonymous point mutations, insertions, deletions, and multiplications. A complete list of mutations and their genetic targets are provided in Additional file 1: Table S1-2. The Quantitative Genomics Facility at ETH Zürich (Basel, Switzerland) performed all of the sequencing. The Genetic Diversity Center at ETH Zürich (Zürich, Switzerland) performed all of the bioinformatics analyses. All of the sequence reads are publically available in the European Nucleotide Archive (http://www.ebi.ac.uk/ena) under accession number PRJEB18464.

### Growth measurements

We measured the time for populations to enter stationary phase by measuring OD_600_ over time. We plated each evolved population on LB agar plates containing 0.1 mM of IPTG, picked ten individual colonies, inoculated the colonies into separate one-mL cultures containing aerobic ACS medium, and let them grow overnight to stationary phase. We then mixed the individual one-ml cultures from each evolved population in equal volumes, inoculated 800 μL of the mixture into 20 mL of dinitrogen gas (N_2_)-sparged ACS medium amended with 10 mM of sodium nitrate (NaNO_3_), and grew the mixture to stationary phase. We used mixtures of ten individual clones from each population to maintain genetic variation of the population. We then inoculated 200 μL of this stationary phase culture into 20 mL of fresh dinitrogen gas (N_2_)-sparged ACS medium amended with 10 mM of sodium nitrate (NaNO_3_) and measured OD_600_ over time with a Synergy plate reader (BioTek, Luzern, Switzerland) until reaching stationary phase.

### Competition assays

We competed a randomly picked clone from each evolved population against the ancestral population that expresses a different fluorescent protein-encoding gene (*i.e*., we competed an *egfp*-expressing evolved clone against the *echerry*-expressing ancestral clone and vice versa). This allowed us to distinguish and quantify the frequencies of different strains when competed against each other. To perform the competition assays, we first plated each evolved population on LB agar plates containing 0.1 mM of IPTG, picked a single random colony from each population, inoculated the colonies into separate one-mL cultures of aerobic ACS medium, and grew them overnight to stationary phase. We then inoculated 800 μL of aerobically grown cells into 20 mL of dinitrogen gas (N_2_)-sparged ACS medium amended with 10 mM of sodium nitrate (NaNO_3_) and grew the cultures until reaching stationary phase. We finally mixed an aliquot of each evolved clone with an aliquot from the ancestral culture (50:50 or 5:95 vol:vol, depending on the experiment). We then inoculated 100 μL of this mixture into 20 mL of fresh dinitrogen gas (N_2_)-sparged ACS medium amended with 10 mM of sodium nitrate (NaNO_3_) and grew the cultures until reaching stationary phase. We plated this mixture onto LB agar plates (two per competition) containing 0.1 mM IPTG prior to incubation and after reaching stationary phase to determine the initial and final frequencies of evolved and ancestral cells. We calculated the fitness of the evolved clones relative to the ancestral clone using equation (1) where W is the relative fitness of the evolved clone, R_0_ is the frequency of evolved cells before competition, R_1_ is the frequency of evolved cells after competition, and F is the fold increase in cell numbers during the competition experiment as determined by the dilution [35, 36].

1 $$ \mathrm{W}=\frac{ \log \left(\frac{{\mathrm{R}}_1\times \mathrm{F}}{{\mathrm{R}}_0}\right)}{ \log \left(\frac{\left(1-{\mathrm{R}}_1\right)\times \mathrm{F}}{1-{\mathrm{R}}_0}\right)} $$

### Biolog assays

We plated each population on LB agar plates containing 0.1 mM IPTG, picked ten individual colonies, inoculated the colonies into separate one-mL cultures of aerobic ACS medium, and let them grow overnight to stationary phase. We then mixed the individual one-ml cultures from each population in equal volumes, diluted the mixture into aerobic ACS medium lacking a carbon source (no citrate or asparagine), and pipetted the mixture into each well of a 96-well PM1 Biolog plate (Biolog, Hayward, CA, USA), where each well contained a different carbon source. We evaluated one PM1 Biolog plate per evolved population and three PM1 Biolog plates for each ancestral population (*i.e*., three PM1 Biolog plates for the *egfp*-containing strain and three PM1 Biolog plates for the *echerry*-containing strain). We incubated the plates at 30 °C with shaking at 220 rpm for 24 h and measured the OD_600_ with an Eon plate reader (BioTek, Luzern, Switzerland) every second hour. We did not use a respiration indicator as is typical for Biolog analyses, and we therefore measured cell density rather than cumulative respiration activity. We did this because cell density provides a more direct measure of growth than does respiration activity. We estimated growth performance for each carbon source provided in the PM1 Biolog plates by calculating the area under the curve (growth over time), which account for both growth rate and yield [22]. We normalized the data by calculating the average for the evolved clone measurements and dividing that value by the average of the ancestor measurements for each carbon source.

### Statistical analyses

We used the non-parametric Wilcoxon rank-sum test to test for differences between different evolution conditions (*i.e*., pH treatments). This test is more robust to outliers than its parametric equivalent, which is particularly relevant to this study. Briefly, we found that one population followed a very different evolutionary trajectory than the other populations (see results section), thus reducing the power of parametric tests and questioning the validity of the normality assumption required by these tests. We tested for differences between groups using analysis of variance (ANOVA) with a subsequent Tukey’s Honest Significant Difference (HSD) post hoc-test. For these analyses, we did not detect any clear outliers, and parametric analyses were therefore deemed appropriate.

## Additional file

Additional file 1: Figure S1. The number of non-synonymous mutations in coding regions in the evolved clones for each experimental evolution condition. Horizontal bars and *P*-values indicate the outcomes from two-sample Wilcoxon rank-sum tests. A star indicates a *P*-value <0.05. Data are presented as Tukey box-plots. **Figure S2.** The relative fitness of the evolved clones calculated from competition with the ancestor at the conditions of the evolution with an inital frequency of 50% of evolved cultures. There is no significant difference in the increase in fitness between pH 7.5 and pH 6.5 (Wilcoxon rank sum test, *P* > 0.4, n_1_ = n_2_ = 8). **Table S1.** Mutations that accumulated in clones evolved at pH 7.5. **Table S2.** Mutations that accumulated in clones evolved at pH 6.5. **Table S3.** Strains and plasmids used in this study. **Table S4.** Oligonucleotide PCR primers used for cloning the *egfp* or *echerry* gene into the pUC18T-mini-Zn7T-LAC-Gm plasmid. (PDF 239 kb)
