# Supplementary material for: Metabolite toxicity determines the pace of molecular evolution within microbial populations
Source: BMC Evol Biol. 2017 Feb 14;17:52. doi: 10.1186/s12862-017-0906-2 (PMC5310025; doi:10.1186/s12862-017-0906-2)
Supplement: Additional file 1: Figure S1. — The number of non-synonymous mutations in coding regions in the evolved clones for each experimental evolution condition. Horizontal bars and P-values indicate the outcomes from two-sample Wilcoxon rank-sum tests. A star indicates a P-value <0.05. Data are presented as Tukey box-plots. Figure S2. The relative fitness of the evolved clones calculated from competition with the ancestor at the conditions of the evolution with an inital frequency of 50% of evolved cultures. There is no significant difference in the increase in fitness between pH 7.5 and pH 6.5 (Wilcoxon rank sum test, P > 0.4, n1 = n2 = 8). Table S1. Mutations that accumulated in clones evolved at pH 7.5. Table S2. Mutations that accumulated in clones evolved at pH 6.5. Table S3. Strains and plasmids used in this study. Table S4. Oligonucleotide PCR primers used for cloning the egfp or echerry gene into the pUC18T-mini-Zn7T-LAC-Gm plasmid. (PDF 239 kb) [file 12862_2017_906_MOESM1_ESM.pdf]

Supplementary Information

Supplementary Figures

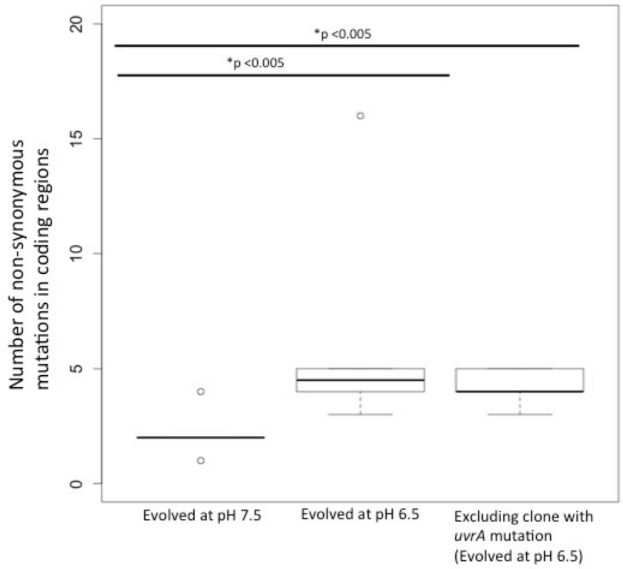

Supplementary Figure S1. The number of non-synonymous mutations in coding regions in the evolved clones for each experimental evolution condition. Horizontal bars and *P*-values indicate the outcomes from two-sample Wilcoxon rank-sum tests. A star indicates a *P*-value < 0.05. Data are presented as Tukey box-plots

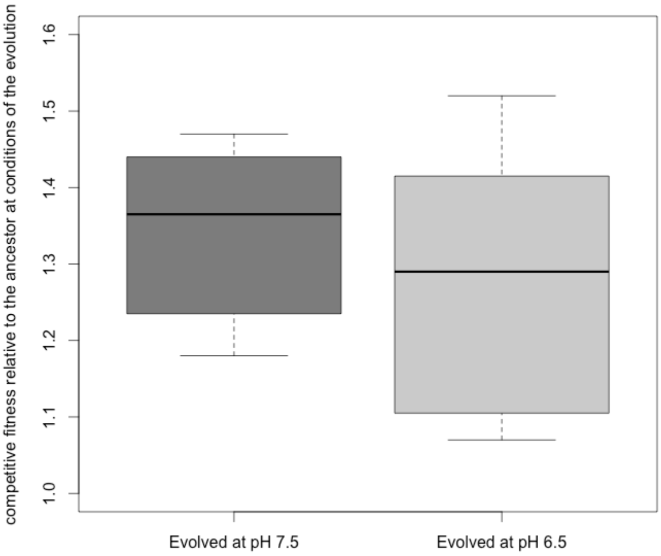

Supplementary Figure S2. The relative fitness of the evolved clones calculated from competition with the ancestor at the conditions of the evolution with an inital frequency of 50% of evolved cultures. There is no significant difference in the increase in fitness between pH 7.5 and pH 6.5 (Wilcoxon rank sum test, $p>0.4$, $n_1=n_2=8$).

Supplementary Tables

Table S1. Mutations that accumulated in clones evolved at pH 7.5.

clone	position	mutation	annotation[a]	gene	description
C1	3,039,855	Δ3bp	coding (194-196/1296 nt)	*oprQ*	outer membrane protein OprE3
C2	1,418,905	A -> C	R401R (CGA -> CGC)	*fsr*	fosmidomycin resistance protein
C2	2,5999,452	Δ12bp	coding (972-983/1293 nt)	*PST_2380*	porin
C2	2,799,697	+C	coding(486/777 nt)	*fliR*	flagellar biosynthesis protein FliR
C3	2,532,546	C -> T	noncoding (1207/2901 nt)	*PST_2318*	23S ribosomal RNA
C3	2,812,145	9bp x 2	duplication	*fliF*	flagellar MS-ring protein
C3	3,392,368	18bp x 2	duplication	*PST_3150*	hypothetical protein
C4	442,947	C -> A	D51Y (GAC -> TAC)	*phoP*	two-component response regulator PhoP
C4	3,392,467	C -> G	G278R (GGC -> CGC)	*PST_3150*	hypothetical protein
D1	1,485,794	Δ1 :: RP10 (-) 1+3bp :: +C	coding (227-229/1317 nt)	*flgE*	flagellar hook protein FlgE
D1	2,599,418	Δ27bp	coding (938-964/1293 nt)	*PST_2380*	porin
D1	3,361,859	Δ1 :: RP10 (-) 1+3bp :: +C	intergenic (+186/-26)	*PST_3122 / PST_3123*	hypothetical protein / general stress protein
D2	2,813,738	RP4 (+) +8np :: Δ1	Intergenic (-21/-75)	*fliE / PST_2592*	Flagellar hook-basal body protein FliE / benzoate transport protein
D2	3,040,582	6bp x 2	duplication	*oprQ*	outer membrane protein OprE3
D2	4,094,823	+G	coding (1241/1452 nt)	*PST_3786*	PAP2 family protein/DedA family protein
D3	442,505	C -> G	R198P (CGT -> CCT)	*phoP*	two-component response regulator PhoP
D3	1,484,866	Δ1 :: RP10 (-) + 3bp :: +C	coding (2-4/681 nt)	*flgD*	basal-body rod modification protein FlgD
D3	2,636,593	A -> G	R702R (CGA -> CGG	*PST_2411*	assimilatory nitrate reductase
D3	3,392,355	+TGA	coding (944/1005 nt)	*PST_3150*	hypothetical protein
D3	4,283,751	Δ84bp	coding (3623-3706/7335 nt)	*PST_3948*	component of chemotactic signal transduction system
D4	1,484,866	Δ1 :: RP10 (-) + 3bp :: +C	coding (2-4/681 nt)	*flgD*	basal-body rod modification protein FlgD
D4	3,392,355	+TGA	coding (944/1005 nt)	*PST_3150*	hypothetical protein

Definitions*:* C clones, clones evolved from the ancestral strain 1601ech. D clones, clones evolved from the ancestral strain 1601gfp.

[a]The type of change that the mutation has caused. D51Y (GAC -> TAC) means that a base pair substitution from G to a T has caused amino acid 51 in this protein to change from aspartic acid (D) to tyrosine (Y). * signifies a stop codon. Intergenic (+186/-26) means that this mutation is located 186 base pairs downstream of one gene and 26 base pairs upstream of another. Coding (972-983/1293 nt) means the mutation is in (between) base pairs 972-983 out of 1293 nucleotides in the coding region of a protein.

Table S2. Mutations that accumulated in clones evolved at pH 6.5.

clone	position	mutation	annotation[a]	gene	description
G1	993,847	G -> A	L178F (CTC -> TTC)	*narX*	two-component sensor NarX
G1	1,260,110	T -> G	T411P (ACC -> CCC)	*pykA*	pyruvate kinase
G1	2,493,376	A -> C	H104P (CAC -> CCC)	*PST_2283*	outer membrane protein
G1	2,599,421	Δ12bp	coding (941-952/1293 nt)	*PST_2380*	porin
G1	2,809,584	C -> T	G78D (GGC ->	*fliI*	flagellum-specific ATP

			GAC		synthase
G2	754,814	+G :: RP10 (+) +3bp :: Δ1	intergenic (+45/-407)	*PST_0656 / PST_0657*	hypothetical protein / transposase like protein TbpA3
G2	1,260,604	A -> G	V246A (GTG -> GCG	*pykA*	pyruvate kinase
G2	2,532,546	C -> T	Noncoding (1207/2901 nt)	*PST_2318*	23S ribosomal RNA
G2	3,040,113	T -> C	L151P (CTG -> CCG)	*oprQ*	outer membrane protein OprE3
G2	3,209,034	T -> G	T53P (ACC -> CCC)	*PST_2974*	3-hydroxyisobutyrate dehydrogenase
G2	2,666,603	C -> T	V801 (GTA -> ATA)	*pilF*	Type IV pilus biogenesis protein pilF
G2	3,818,939	Δ1bp	intergenic (-17/+53)	*nirT / nirS*	tetraheme protein nirT / cytochrome cd1 nitrite reductase
G3	379,263	G -> A	G51D (GGC -> GAC)	*fbp*	fructose- 1,6 - biphosphatase
G3	442,411	Δ408bp		*phoP – phoQ*	
G3	2,415,795	T -> C	intergenic (-1849/-67	*PST_2210 / PST2213*	udecaprenyl pyrophosphatase / hypothetical protein
G3	2,771,593	T -> C	D338G (GAC -> GGC)	*fleQ*	transcriptional regulator FleQ
G3	3,040,113	T -> C	L151P (CTG -> CCG)	*oprQ*	outer membrane protein OprE3
G3	4,056,643<br>4,057,686	RP3 (+) Δ1bp<br>Δ66bp	intergenic (+170/+2139) intergenic (+1213/+1029)	*PST_3751 / PST_3752*	CRISPR-associated Cas2 family protein / hypothetical protein
G4	379,170	C -> G	P20R (CCG -> CGG)	*fbp*	fructose- 1,6 - bisphosphatase
G4	995,501	G -> C	G286R (GGC -> CGC)	*narK*	nitrite extrusion protein
G4	2,532,535<br>2,532,546<br>2,532,548	G -> C<br>C -> T<br>C -> G	noncoding (1218/2901 nt) (1207/2901 nt) (1205/2901 nt)	*PST_2318*	23S ribosomal RNA
G4	3,040,113	T -> C	L151P (CTG -> CCG)	*oprQ*	outer membrane OprE3
H1	442,210	C -> G	A93P (GCC-> CCC)	*phoQ*	two-component sensor PhoQ
H1	881,077	C -> T	A432V (GCG -> GTG)	*rpoB*	DNA-directed RNA polymerase subunit beta
H1	1,926,532	G -> T	S508Y (TCC -> TAC)	*xcpQ*	general secretion pathway protein D
H1	2,796,711	G -> A	Q223* (CAG ->TAG)	*flhA*	flagellar biosynthesis protein FlhA
H1	2,875,014	G -> A	P24L (CCG -> CTG)	*gap-2*	glyceraldehyde-3-phosphate dehydrogenase
H1	4,057,221	Δ67bp	intergenic (+748/+1493)	*PST_3751 / PST_3752*	CRISPR-associated Cas2 family protein / hypothetical protein
H2	17,482	A -> G	V195A (GTC -> GCC)	*PST_0015*	amino acid ABC transporter permease
H2	197,672	G -> A	A64T (GCC -> ACC	*ohr*	organic hydroperoxide resistance protein
H2	284,771	C -> T	G508D (GGT -> GAT)	*PST_0249*	hypothetical protein
H2	318,618	C -> T	L353L (CTG -> TTG)	*argA*	N-acetylglutamate synthase
H2	379,637	C -> T	L176F (CTT -> TTT)	*fbp*	fructose-1,6-bisphophatase
H2	712,930	G -> A	P8S (CCA -> TCA)	*PST_0627*	hypothetical protein
H2	810,646	RP6(-)+7bp	intergenic (+55/+33)	*PST_0704 / PST_0705*	hypothetical protein / hypothetical protein
H2	850,146	C -> T	G96G (GGC -> GGT)	*gph*	phosphoglycolate phosphatase

H2	876,102	C -> T	intergenic (+9/-43)	*tuf / PST_0770*	elongation factor Tu / tRNA-Trp
H2	910,678	RP6 (+) +7bp	coding (364-370/2958nt)	*uvrA*	excinuclease ABC subunit A
H2	1,260,392	C -> T	A317T (GCG -> ACG)	*pykA*	pyruvate kinase
H2	1,510,239	RP6(-)+6bp	coding (726-731/1773nt)	*PST_1412*	EAL/GGDEF domain-containing protein
H2	1,575,460	G -> A	K89K (AAG -> AAA)	*PST_1469*	type I restriction-modification system, M subunit
H2	1,823,732	G -> A	L383L (CTG -> CTA)	*PST_1694*	acyl-CoA dehydrogenase
H2	1,948,354	Δ1bp	coding (600/1440nt)	*cydA2*	cytochrome d ubiquinol oxidase, subunit I
H2	2,332,524	G -> A	R259Q (CGG -> CAG)	*PST_2148*	hypothetical protein
H2	2,404,122	C -> T	C3C (TGC -> TGT)	*PST_2202*	hypothetical protein
H2	2,532,546	C -> T	noncoding (1207/2901nt)	*PST_2318*	23S ribosomal RNA
H2	2,812,452	T -> C	E299G (GAG -> GGG)	*fliF*	flagellar MS-ring protein
H2	3,039,855	Δ3bp	coding (194-196/1296nt)	*oprQ*	outer membrane protein OprE3
H2	3,086,463	G -> A	F89F (TTC -> TTT)	*PST_2869*	gamma-glutamyktranspeptidase
H2	3,495,820	G -> A	R423C (CGC -> TGC)	*PST_3235*	alpha,alpha-trehalose-phosphate synthase
H2	3,590,596	C -> T	G131D (GGT-> GAT)	*folP*	dihydropteroate synthase
H2	3,937,070	T -> C	D282G (GAC -> GGC)	*PST_3647*	hypothetical protein
H2	3,942,203	C -> T	R281R (CGG -> CGA)	*PST_3650*	hypothetical protein
H2	4,391,436	C -> T	S34F (TCT -> TTT)	*tctD*	transcriptional regulatory protein TctD
H3	379,227	A -> G	H39R (CAC -> CGC)	*fbp*	fructose-1,6-bisphosphatase
H3	442,534	G -> C	N188K (AAC -> AAG)	*phoP*	two-component response regulator PhoP
H3	2,772,535	Δ1bp	coding (71/1515nt)	*fleQ*	transcriptional regulator FleQ
H3	3,040,113	T -> C	L151P (CTG -> CCG)	*oprQ*	outer membrane protein OprE3
H4	379,374	C -> T	A88V (GCT ->GTT)	*fbp*	fructose-1,6-bisphosphatase
H4	2,771,479	T -> C	N376S (AAC -> AGC)	*fleQ*	transcriptional regulator FleQ
H4	3,040,113	T -> C	L151P (CTG -> CCG)	*oprQ*	outer membrane protein OprE3
H4	3,993,470	G -> A	P668S (CCG -> TCG)	*PST_3693*	tetrathionate reductase subunit A
H4	4,231,226	T -> C	I18V (ATT -> GTT)	*PST_3901*	hypothetical protein

Definitions*:* G clones, clones evolved from the ancestral strain 1601ech. H clones, clones evolved from the ancestral strain 1601gfp.

[a]The type of change that the mutation has caused. L178F (CTC -> TTC) means that a base pair substitution from C to a T has caused amino acid 178 in this protein to change from leucine (L) to phenylalanine (F). * signifies a stop codon. Intergenic (+45/-407) means that this mutation is located 45 base pairs downstream of one gene and 407 base pairs upstream of another. Coding (941-952/1293 nt) means the mutation is in (between) base pairs 941-952 out of 1293 nucleotides in the coding region of a protein

Table S3. Strains and plasmids.

Strain or plasmid	Relevant characteristics	Reference or source
***P. stutzeri* strain**		
A1601gfp	A1501 with Δ*comA* and mini-Tn7T-LAC-Gm-*egfp*; Gm^R, $egfp^+$	This study
A1601ech	A1501 with Δ*comA* and mini-Tn7T-LAC-Gm-*echerry*; Gm^R, $echerry^+$	This study
***E. coli* strain**		
DH5α/λpir	Used for replication of pUC18T and pAW19 derivatives; λ*pir*80*dlacZ*Δ*M15* Δ(*lacZYA-argG*)*U169 recA1 hsdr17 deoR thi-1 supE44 gyrA96 relA*	(Miller and Mekalanos, 1988)
SM10/λpir	Used for replication of pUX-BF13; *thi-1 thr leu tonA lacY supE recA*::RP4-2-Tc::Mu, Km^R, λ*pir*	(Miller and Mekalanos, 1988)
BW20767	Used for conjugative transfer of pAW19 derivatives; RP4-2-Tc::Mu-1 Kan::Tn*7* integrant *leu-63*::IS*10 recA1 zbf-5 creB510 hsdR17 endA1 thi uidA* (ΔMluI)::pir^+	(Metcalf *et al.*, 1995)
**Plasmid**		
pUC18T-mini-Tn7T-LAC-Gm	pUC18-based conditionally replicative delivery plasmid for mini-Tn7-LAC-Gm; Ap^R, Gm^R, mob^+	(Choi *et al.*, 2005)
pUC18T-mini-Tn7T-LAC-Gm-e*gfp*	pUC18T-mini-Tn7T-LAC-Gm containing e*gfp* immediately downstream of P_{lac}; Ap^R, Gm^R, $mob^{+,}$ $egfp^+$	This study
pUC18T-mini-Tn7T-LAC-Gm-e*cherry*	pUC18T-mini-Tn7T-LAC-Gm containing e*cherry* immediately downstream of P_{lac}; Ap^R, Gm^R, $mob^{+,}$ $echerry^+$	This study
pUX-BF13	R6K replicon-based helper plasmid that provides the Tn7T transposition function in *trans*; Ap^R, mob^+	(Bao *et al.*, 1991)

Table S4. Oligonucleotide PCR primers used for cloning the egfp or echerry gene into the pUC18T-mini-Zn7T-LAC-Gm plasmid.

Gene	Direction	[a]Primer sequence (5'-3')	Restriction site
egfp	Forward	CGCGGATCCTGATTAACTTTATAAGGAGGAAAAAC ATATGAGTAAAGGAGAAGAACTTTTCACT	BamHI
	Reverse	CGGGGTACCTTTGTATAGTTCATCCATGCCATG	KpnI
echerry	Forward	CGCGGATCCTGATTAACTTTATAAGGAGGAAAAAC ATATGGTTTCCAAGGGCGAG	BamHI
	Reverse	CGGGGTACCTTATTTGTACAGCTCATCCATGC	KpnI

[a]Red: leader sequence for restriction digestion. Blue: restriction sequence. Green: ribosomal binding sequence. Black: target-specific sequence.
